# A severe case of human rhinovirus A45 with central nervous system involvement and viral sepsis

**DOI:** 10.1186/s12985-022-01799-x

**Published:** 2022-04-22

**Authors:** Jun Liu, Hongwei Zhao, Ziheng Feng, Yingchao Liu, Qianyu Feng, Suyun Qian, Lili Xu, Hengmiao Gao, Zhengde Xie

**Affiliations:** 1 Beijing Key Laboratory of Paediatric Respiratory Infection Diseases, Key Laboratory of Major Diseases in Children, Ministry of Education, National Clinical Research Center for Respiratory Diseases, National Key Discipline of Paediatrics (Capital Medical University), National Clinical Research Center for Respiratory Diseases, Beijing Paediatric Research Institute, Department of Paediatric Critical Care Medicine, Beijing Children’s Hospital, Capital Medical University, National Center for Children’s Health, Beijing, China; 2 Research Unit of Critical Infection in Children, Chinese Academy of Medical Sciences, Beijing, 2019RU016 China

**Keywords:** Rhinovirus, Central nervous system involvement, Viral sepsis

## Abstract

**Background:**

Rhinovirus is a common viral aetiology of upper respiratory infection and is mostly associated with common cold or flu-like illness. Although rhinovirus has been recognized as a pathogen for lower respiratory infections in severe cases credited to advances in molecular detection, central nervous system involvement and multiorgan dysfunction are extremely rare.

**Case presentation:**

A previously healthy 10-year-old girl developed fever, sore throat and conjunctive injection after contact with an upper respiratory infection patient, followed by seizures, haematuria, and severe diarrhoea. She experienced viral sepsis and multiorgan dysfunction after admission. Cerebral computed tomography showed significant diffuse encephaledema. Cerebrospinal fluid analysis showed significantly elevated protein levels. After her consciousness disturbance improved, she still took a long time to recover from haematuria and diarrhoea. We identified a rarely reported rhinovirus A45 in her oropharyngeal and anal swabs by metagenomic next-generation sequencing, and bacterial culture of blood specimens yielded negative results.

**Conclusions:**

This case presents a patient with severe rhinovirus infection, which was very likely responsible for her central nervous system symptoms and viral sepsis.

## Background

Human rhinovirus (HRV) is classified in the *Enterovirus* genus within the *Picornaviridae* family. It is a common viral aetiology of upper respiratory infections and has been mostly associated with cold and flu-like illnesses [1]. In most cases, HRV infection is mild and self-limited, but it is responsible for severe symptoms in children, such as wheezing, exacerbation of asthma, bronchiolitis, severe pneumonia, and cardiopulmonary disease [2–4]. Risk factors associated with moderate to severe HRV infections include comorbidities and RSV coinfection, prematurity, congenital heart diseases, and noninfectious respiratory diseases (including obstructive sleep apnea, asthma, chronic obstructive pulmonary disease, bronchiectasis, interstitial and pulmonary vascular diseases, etc.) [5].

Enterovirus (enterovirus-A71, enterovirus-D68, type B coxsackieviruses, etc.) is frequently considered to be the most likely pathogen by paediatricians in cases of aseptic meningitis, encephalitis, encephalopathy, and disseminated infection in children [6, 7]. However, HRV, also a member of enterovirus genus, is rarely associated with central nervous system (CNS) infection. To date, only a few cases of HRV-related encephalitis/encephalopathy have been reported worldwide [8–12].

Herein, we presented a severe case of HRV-related CNS involvement in a previously healthy child without known medical history. HRV infection was confirmed by metagenomic next-generation sequencing (mNGS) and virus isolation.

## Case presentation

In this case, the patient was a 10-year-old girl who was born full-term and developed normally. She had no recorded medical issues of congenital heart diseases or noninfectious respiratory disease. There were no noteworthy diseases in her family history, except that her mother had a miscarriage at 20 weeks gestation in her third pregnancy. She has a healthy twin brother.

On the 10th of July (five days before admission), the patient experienced sore throat, redness in her eyes, and neck stiffness and developed fever with a temperature up to 40.5 °C after contact with a patient with a respiratory infection (her mother). She was prescribed azithromycin, but it did not relieve the symptoms. She was presented to the emergency department after an episode of convulsion and loss of consciousness lasting 2 min and ceased spontaneously. The second episode of convulsion occurred when she was observed in the emergency room, which could not be controlled by diazepam (10 mg) and chloral hydrate (10 ml). She did not regain consciousness after this episode and bloody urine could be seen in the catheter, wherefore was admitted to a paediatric intensive care unit (PICU).

**Fig. 1:**
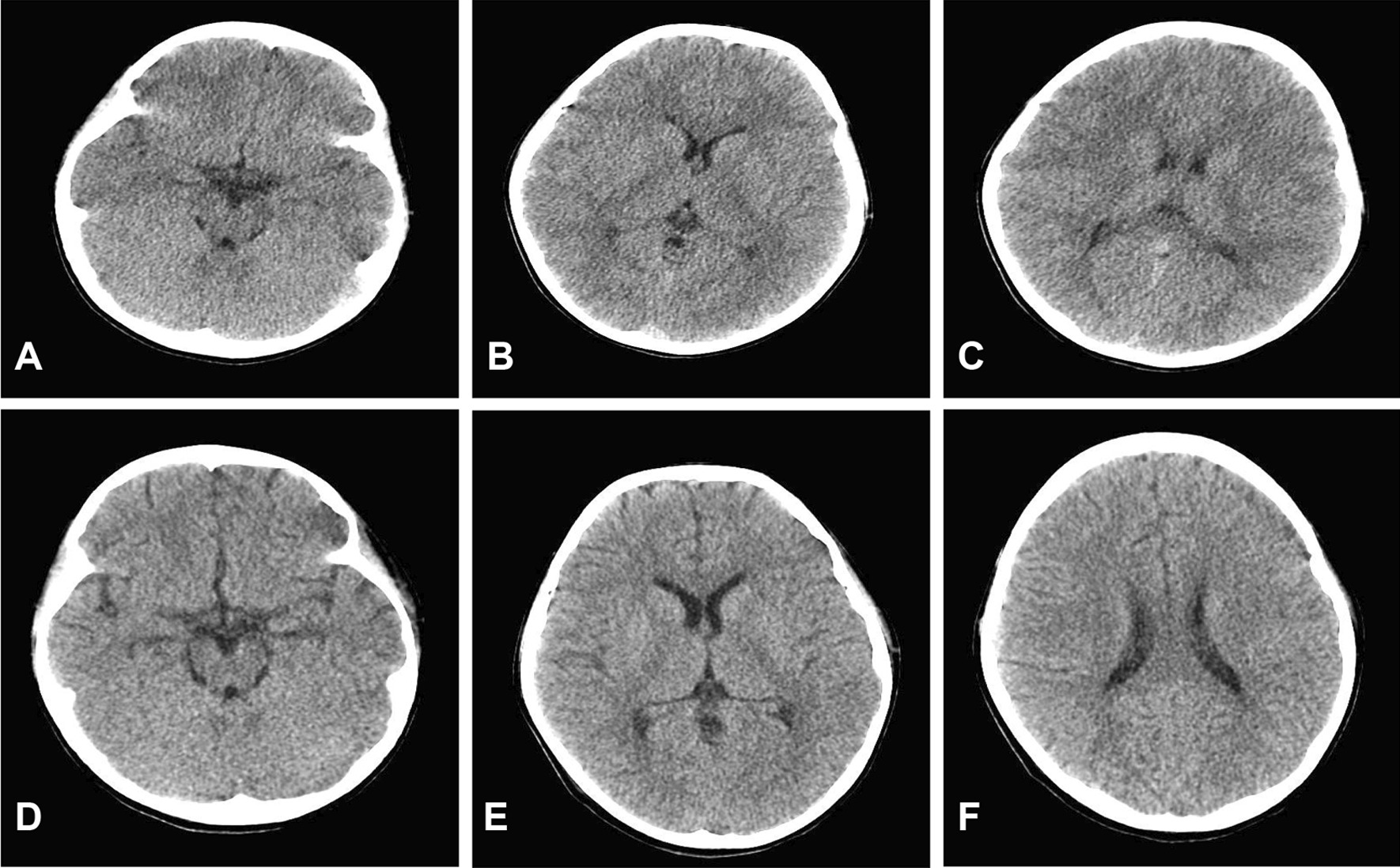
Cerebral computed tomography results. **A**–**C** Images obtained on admission Day 1 show a finding suggestive of diffuse encephaledema. **D**–**F** Images obtained on admission Day 7 show improvement of encephaledema

On examination, her temperature was 36.5 °C, her blood pressure was 134/89 mmHg, her heart rate was 172 beats per minute, her respiratory rate was 37 breaths per minute, and her oxygen saturation was 96% while the patient was breathing ambient air. Her Glasgow Coma Scale (GCS) score was 6 (E1V1M4). Her pupillary size was 3 mm in both eyes, but the pupillary response was delayed, and conjunctives were injected and oedematous. Neither meningeal nor abnormal neurologic signs were noted. On abdomen auscultation, bowel sounds decreased. Other physical findings were unremarkable. Her head computed tomography showed significant diffuse encephaledema (Fig. 1A–C). Laboratory tests were in the normal range except for slightly elevated procalcitonin (PCT) levels.

**Fig. 2:**
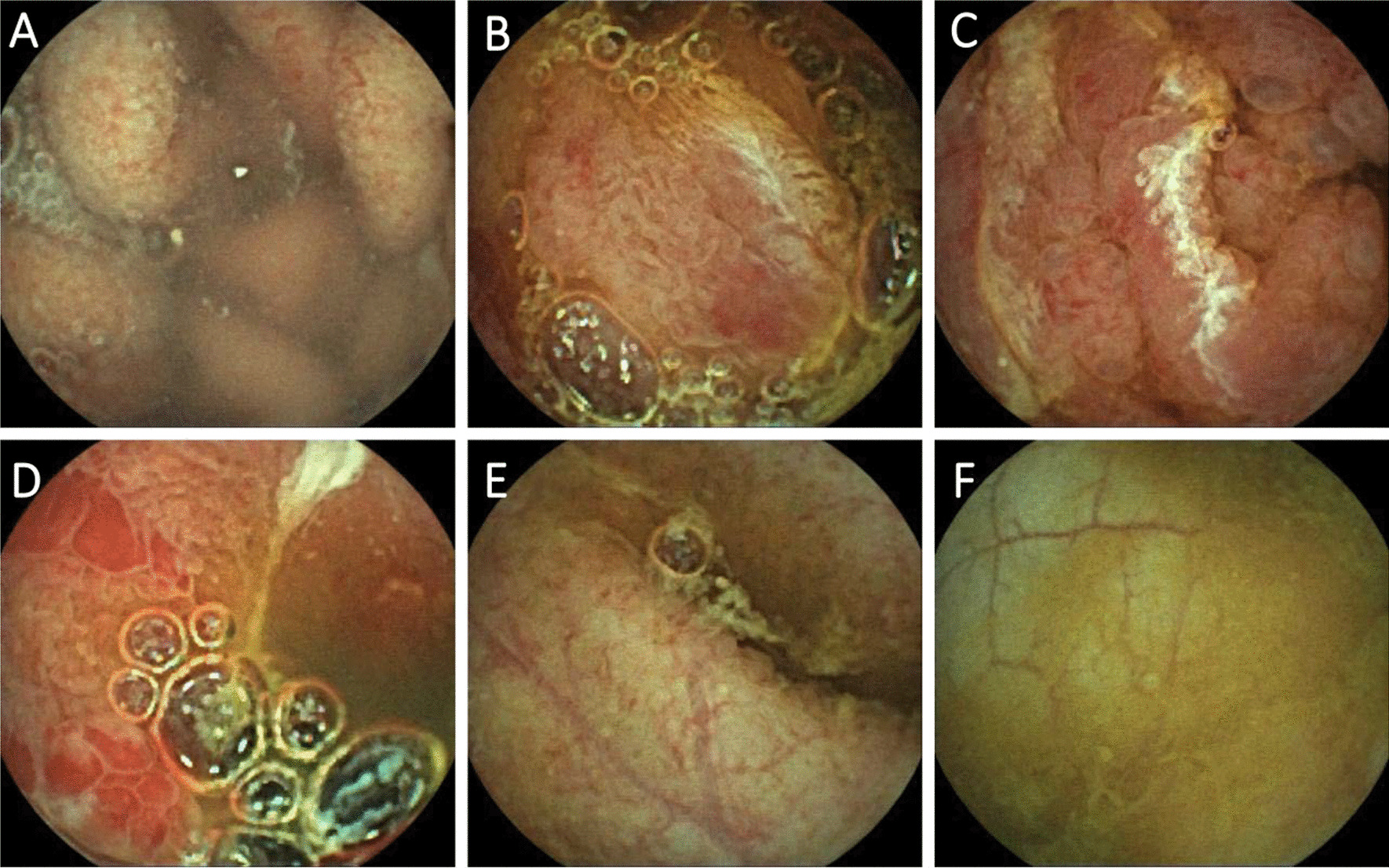
Images of video capsule endoscopy on hospital Day 37. **A** Gastric antrum; **B** duodenum; **C** jejunum; **D** ileum; **E** terminus of ileum; **F** colon

The patient received standard supportive care after admission, including Nasal Continuous Positive Airway Pressure (NCPAP), continuous intravenous midazolam, methylprednisolone (2 mg/kg·d), mannitol, and 3% saline. Six hours after transfer to the PICU, the patient underwent intubation and mechanical ventilation for paradoxical respiration and worsening oxygen saturation with cyanosis. The patient exhibited significant elevations of creatine kinase (1345 U/L; reference range, 25–200 U/L) and myoglobin (247.8 ng/ml; reference range, 0–140 ng/ml), and the diagnosis of rhabdomyolysis was confirmed accordingly. Other enzymes were measured, including alanine transaminase, aspartate transaminase, pancrelipases, pancreatic amylase, amylase, lactate dehydrogenase, which all were elevated and details were listed in Table 1. Detailed laboratory results were also listed in Table 1. On hospital Day 2, cerebrospinal fluid (CSF) analysis revealed that protein levels were significantly increased by nearly 3 times the upper limit of the reference range (1288 mg/L; reference range, 20–450 mg/L), and glucose levels were slightly elevated (6.29 mmol/L; reference range, 2.8–4.5 mmol/L), while Gram staining of the CSF was negative. Considering autoimmune encephalitis, intravenous immunoglobulin (IVIG) was administered. On hospital Day 3, the patient’s situation deteriorated, and she developed hypotension and hypovolemia. Blood analysis revealed decreased WBC counts and elevated C-reactive protein levels (CRP). Her PCT level was 157.97 ng/L. Cytokine levels were examined, and the results showed elevated levels of IL-6 (388.67 pg/ml [reference range < 5.40 pg/ml]), IL-8 (1116.66 pg/ml [reference range < 20.60 pg/ml]), IL-10 (17.88 pg/ml [reference range < 12.90 pg/ml]), and IFN-γ (38.21 pg/ml [reference range < 23.10 pg.ml]). In combination of her laboratory results, proven pathogen infection by PCR assay and clinical manifestation, she was diagnosed with septic shock, and anti-shock therapy was initiated. Meropenem and linezolid were prescribed to control an infection. Doses of methylprednisolone were increased to 10 mg/kg. On physical examination, her pupils were dilated, and pupillary light reflexes were prolonged, findings suggestive of intracranial hypertension. Because the patient had progressively increased creatine levels and mixed metabolism and respiratory acidosis, continuous renal replacement therapy (CRRT) with continuous venovenous haemofiltration was started. On hospital Day 4, she developed coagulation disorder and pancytopenia. Haemophagocytic syndrome was considered, but the laboratory results did not meet the criteria to support the diagnosis until hospital Day 17 (pancytopenia, elevated serum ferritin [777.4 ng/ml, reference range 6–159 ng/ml], SCD25, decreased NK cell counts and phagocytosis in bone marrow). Bloody fluid was visible in the indwelling gastrointestinal decompression tube with decreased haemoglobin levels of 7.1 g/dl, and the patients had bloody and watery diarrhoea, which prompted active gastrointestinal bleeding. After medical interventions and supportive care, the symptoms of intracranial hypertension were mitigated, and the patient’s awareness improved on hospital Day 6. Computed tomography shows improvement of encephaledema (Fig. 1D–F). We monitored her blood enzymes, which returned to the normal range. On hospital Day 12, the patient was awake, and spontaneous breathing was restored. Levels of CRP and PCT dropped to the reference range. CSF analysis showed normal cell count and chemistries. However, the patient still experienced persistent diarrhoea and haematuria. When her gastrointestinal condition improved, we performed video capsule endoscopy on hospital Day 37, uncovering diffuse intestinal abnormalities with a gross appearance characterized by continuous lesions, ulcerations, erythema, oedema, and inflammatory hyperplasia (Fig. 2). We performed endoscopy on hospital Day 44, and endoscopic biopsy suggested diffuse nonspecific inflammation of the intestine. Haematuria stopped on hospital Day 42. The patient had gradual improvement in digestive tract function, and diarrhoea mitigated gradually. She could tolerate enteral nutrition on hospital Day 38, and parenteral nutrition was stopped on hospital Day 51. She recovered well. After 75 days in hospital, she was discharged.

**Table 1:** Patient laboratory data

Variable	On admission	Hospital day 3	Hospital day 12	Reference range, children*
Haematocrit (%)	38.1	30.8	16.2	36–46
Haemoglobin (g/L)	134	109	59	118–156
White blood cells (×10^9^/L)	14.39	2.77	7.23	4.3–11.3
Differential count (per µl)				
Neutrophils	13.16	2.27	4.79	1.6–7.8
Neutrophils (%)	91.4	82	66.2	31–70
Lymphocytes	0.69	0.4	1.27	1.5–4.6
Lymphocytes (%)	4.8	14.4	17.6	23–59
Platelets (×10^12^/L)	200	120	66	167–453
Prothrombin time (sec)	–	> 300	16.8	14.3–18.8
Activated partial-thromboplastin time (sec)	–	> 180	32.9	25.1–38.4
Prothrombin-time international normalized ratio	–	1.48	1.07	
Total bilirubin (mmol/L)	49.7	56.15	170.59	3.42–20.5
Direct bilirubin (µmol/L)	38.87	47.87	129.84	0–3.42
Indirect bilirubin (µmol/L)	10.2	8.28	40.75	0–17.10
Alanine transaminase (U/L)	94.1	52.4	58.2	7–30
Aspartate transaminase (U/L)	111.5	120.2	48.1	14–44
Pancrelipases (U/L)	1683	2810	106	0–125
Pancreatic amylase (U/L)	27	94.4	82.2	0–39
Amylase (U/L)	184	232	63	17–115
Creatine kinase (U/L)	1345	1551	105	25–200
Lactate dehydrogenase (U/L)	620	648	392	110–295
C-reactive protein (mg/L)	14	154	57	< 8
Procalcitonin (ng/mL)	19.78	157.97	0.77	< 0.25
Blood urea nitrogen (µmol/L)	12.15	13.29	10.15	2.5–6.5
Creatine (µmol/L)	93.6	159.1	55.4	27–66
Myoglobin (ng/mL)	247.8	–	77.2	0–140

^*^Reference values are affected by many variables, including the patient population and the laboratory methods used. The ranges used at Beijing Children’s Hospital are for children. They may, therefore, not be appropriate for all patients

–The laboratory test was not performed on that day

### Viral analysis

#### PCR assay

Viral detection for human enterovirus (HEV) assay targeting the 5′-untranslated region was conducted using real-time reverse transcription polymerase chain reaction (PCR) by pan-enterovirus detection kit (DAAN GENE, Cat. #DA-BT109). Throat swabs and anal swabs were positive, whereas CSF specimens (collected on the day of admission) were negative.

#### mNGS for pathogen detection

Oropharyngeal swabs, anal swabs, and serum specimens (collected on hospital Day 4) were subjected to metagenomic analyses using an NGS platform. Nucleic acids were extracted from each sample using the Direct-zol RNA miniprep kit (Zymo Research, Irvine, CA, USA) and TRIzol LS reagent (Thermo Fisher Scientific, Carlsbad, CA, USA). The DNA/RNA concentrations were measured using a Qubit fluorometer (Thermo Fisher Scientific). DNA libraries were constructed through transposase mediated methods, and PCR amplification followed (Life Technologies, Carlsbad, CA). The quality of the DNA/RNA libraries was assessed using a Qsep1 biofragment analyser (BiOptic, La Canada Flintridge, CA) to measure the sizes of fragments before sequencing. The quantified libraries were pooled and sequenced on a NextSeq 550Dx sequencing platform (Illumina). A single-end (75-bp read length) sequencing strategy was used. Reads that passed these filters were mapped against human references using Bowtie v2.2.4. Reads that aligned to either of the references were removed. The remaining reads were subjected to a BLASTn v2.2.30 search, and mapping was performed using CLC Genomics Workbench v21.0.3. All tools were run with default parameters unless otherwise specified. Human rhinovirus type A (HRV A) was identified from oropharyngeal swabs (3274 reads) and anal swabs (9 reads). These random distributions of reads of HRV A sequences covered 100% of the total HRV A genome. GB virus C (hepatitis G virus) sequences (1091 reads) were detected in blood samples, but we considered it to be the causative pathogen because GB virus C single infection barely had clinical implications. An abundance of sequences of conditional pathogenic bacteria were detected in oropharyngeal swabs and anal swabs, including *Streptococcus anginosus*, *Prevotella melaninogenica*, *Veillonella parvula*, *Bacteroides thetaiotaomicron,* and *Klebsiella pneumoniae*. Large amounts of *Streptococcus anginosus* reads in DNA (up to 18,274) and RNA (up to 413,806) libraries were detected in oropharyngeal swabs and anal swabs, and we suspected it could be a co-pathogen. However, cultures of blood samples yield negative results and the patient’s clinical presentation didn’t warrant a bacterial infection.

#### Viral molecular analysis

**Fig. 3:**
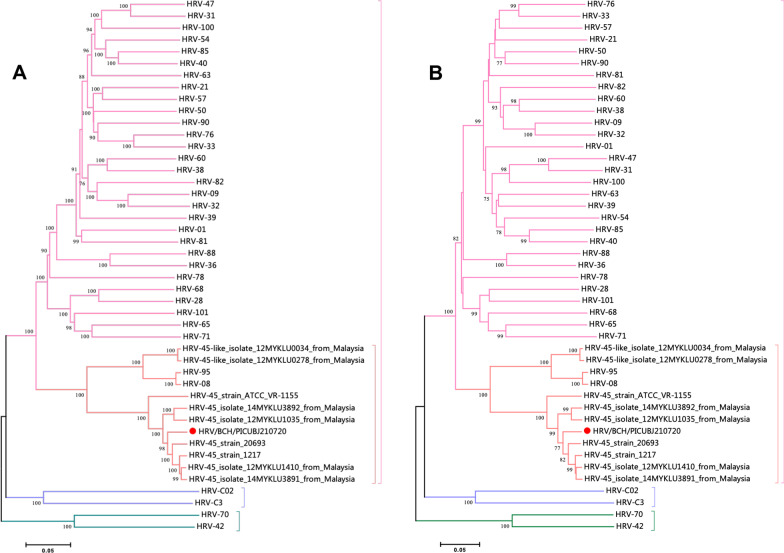
Phylogenic analysis of HRV-A45 detected in this patient and other representative sequences of HRV. MEGA v7.0 software was used to generate phylogenetic trees with the neighbour-joining method and the Kimura 2-parameter model. The robustness of the phylogenetic trees was assessed using the bootstrap method with 1,000 replicates. The strain in the current study is marked with a solid red circle. **A** Phylogenic tree based on the completed genome sequences; **B** phylogenic tree based on the VP1 gene sequences; **C** phylogenic tree based on the 5’UTR sequences

Conventional reverse transcriptase PCR (RT–PCR) targeting the VP4/VP2 region of HRV-A was used to verify the mNGS results as described elsewhere [13]. The complete length of the HRV A genome was obtained using the NGS method. This HRV strain was subtyped as HRV-A and named HRV/BCH/PICUBJ210720. The complete genome sequence was deposited in GenBank (accession number: OL989374). Multiple-sequence alignments were constructed with MAFT (https://www.ebi.ac.uk/Tools/msa/mafft/) software (version 7.407) using the accuracy-oriented method (L-INS-i). Phylogenetic trees based on the complete genomes and viral gene VP1 were generated using the neighbour-joining method, and bootstrap values of MEGA software version 7 with 1000 replicates were calculated to evaluate confidence estimates (Fig. 3). Phylogenetic analysis showed that the HRV-A strain in this study was subgrouped into A45. Nucleotide homology comparisons indicated that this strain was closely related to four strains (HRV-45_strain_20693, HRV-45_strain_1217, HRV-45_isolate_12MYKLU1410_from_Malaysia, HRV-45_isolate_14MYKLU3891_from_Malaysia), which were denoted as rhinovirus A clade D [14]. Compared to the HRV-45 prototype strain ATCC VR-1155, 51 amino acid missense mutations were identified in HRV/BCH/PICUBJ210720. Two unreported substitutions, C900H in P2-A and N2096T in P3-C, were found in this virus. Notably, two substitutions, K654T in the BC loop of VP1 and Q228K in the puff region (EF loop) of VP2, have been reported to associate with viral neutralization sites and virulence [15].

#### Virus isolation

**Fig. 4:**
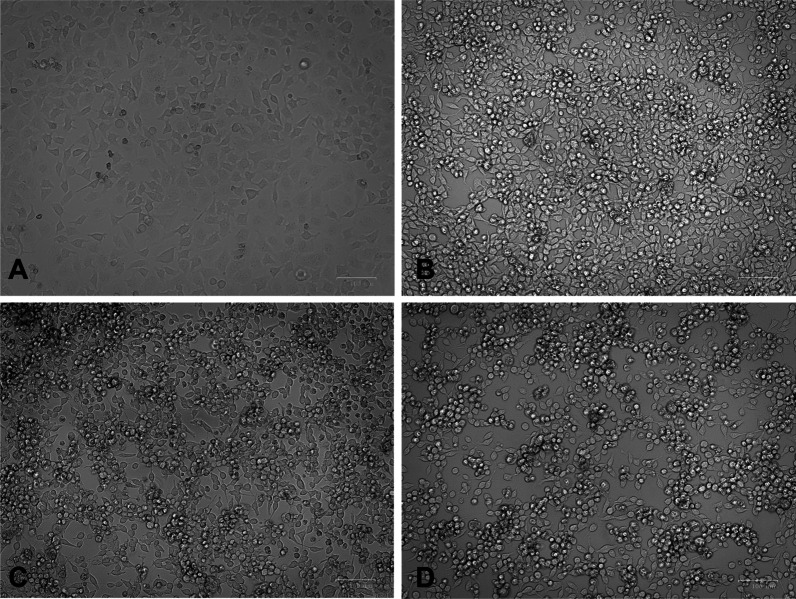
Cytopathic effect (CPE) in H1-HeLa cells after inoculation and 5 days post-inoculation of clinical specimens. **A** Control 0 days after inoculation with DMEM; **B** control 5 days post-inoculation with DMEM; **C** 5 days after inoculation with oropharyngeal swab; **D** 5 days after inoculation with anal swab

Virus was isolated from oropharyngeal swab samples by growing in H1-HeLa cells. Cell lines were cultured at 37 °C in 5% carbon dioxide in Dulbecco's modified Eagle’s medium (DMEM, Gibco) with 10% foetal bovine serum (FBS, Gibco) and 1% penicillin and streptomycin (P/S, Gibco). The sample was centrifuged at 10,000 rpm for 20 min at 4 °C, and 100 µl of the supernatant in 200 µl DMEM supplemented with 1% P/S was inoculated onto monolayers of H1-HeLa cells, which were grown in 24-well cell culture plates after removing the growth medium and washed with DMEM. The cells were incubated for 1 h at 33 °C to allow virus absorption. Then, the inoculum was removed, and DMEM supplemented with 2% FBS and 1% P/S was added to each well. The cultures were incubated further in a 5% CO_2_ incubator at 33 °C. Cytopathic effects (CPEs) were observed on Day 5 after inoculation (Fig. 4).

### Whole exome sequencing

DNA was isolated from peripheral blood samples obtained from the patient using a Gentra Puregene Blood Kit (QIAGEN, Hilden, Germany). The SureSelect Human All Exon Kit (Agilent Technologies, Santa Clara, America) was used for whole exome capture. The target regions were sequenced on NovaSeq (Illumina, San Diego, America) and aligned to the GRCh37/hg19 human reference sequence. Variants were annotated and filtered by TGex (tgex-app.genecards.cn). Variants were classified following the ACMG/AMP interpretation standards and guidelines [16]. Whole-exome sequencing revealed heterozygous mutation of ALMS1 related to Alstrom Syndrome, which was characterized by a complex array of clinical presentations, including progressive retinal degeneration, hearing loss, hyperinsulinaemia, dilated cardiomyopathy, and fibrosis. However, these symptoms did not match the clinical manifestations of our patient.

## Discussion

Human enteroviruses (EVs) are divided into seven species, including enterovirus species A to D and rhinovirus species A to C [17]. EVs have been strongly associated with neurotropic pathogens causing a wide spectrum of clinical manifestations in the CNS ranging from aseptic meningitis to severe encephalitis/encephalopathy. Some species, such as EV-A71, CV-A6, CV-A16, and EV-D68, have been shown to be strongly neurotropic and responsible for diverse neurological complications [18]. However, HRV typically causes symptoms, such as fever, rhinitis, and cough, which are characterized as common cold or flu-like illnesses. HRV-related CNS involvement and multiorgan dysfunction are extremely rare. In this report, we presented a previously healthy girl who developed fever and sore throat, followed by seizures, haematuria, and severe diarrhoea, and experienced viral sepsis and multiorgan dysfunction after admission. We identified a rarely reported HRV-A45 in her oropharyngeal and anal swabs by mNGS. Besides HRV-A45, we also detected a large amount of *Streptococcus anginosus* reads in in oropharyngeal swabs and anal swabs. However, the patient’s clinical presentation didn’t warrant a bacterial infection. Also, we empirically used antibiotics (meropenem and linezolid) to control infection, but they didn’t relieve the patient’s symptoms. Most importantly, bacterial culture of blood specimens yielded negative results. Thus, we presumed that *Streptococcus anginosus* might not attribute to the patient’s clinical presentation.

Viral sepsis is defined as life-threatening organ dysfunction due to an inappropriate, severe inflammatory response in the host to suspected or confirmed viral infection [19, 20]. For clinicians, septic patients lacking evidence of bacterial or fungal infections should always consider viral sepsis. In clinical practice, bacterial sepsis is presumably more common, and antibiotics are administered in most septic patients. Virus tests and identification are frequently foregone in the acute presentation of sepsis. However, in a global epidemiological study of paediatric severe sepsis, 21% of patients had confirmed viral infection, whereas bacteria and fungi made up 54.4% and 13.4% of infectious aetiologies, respectively [21]. Of these viral infection cases, rhinovirus, respiratory syncytial virus, and adenovirus are most commonly identified. In our case, clinical presentations, laboratory tests, and metagenomic analysis all prompted the diagnosis of viral sepsis caused by HRV infection.

HRV is a positive-sense, single-stranded RNA (ssRNA) virus that can be recognized by Toll-like receptors (TLRs). Once double-stranded RNA is generated, it can be recognized by melanoma differentiation-associated gene 5 (MDA-5) and subsequently activate the type I interferon response. The engagement of these receptors is essential to employ the innate immune system to trigger inflammation and antiviral responses [22]. The interaction between these ligands and receptors is required for maximizing HRV-induced IFN-γ, IFN-β, and proinflammatory cytokine expression, including IL-6, IL-8, and IP-10 [23]. As a result, when the immune system tries to eradicate invading pathogens, the virus can utilize these strategies to cause an exaggerated inflammatory response and tissue damage. In this case, they are critical to the ability to cause viral sepsis, which leads to multiorgan damage or dysfunction. We observed significantly elevated IL-6, IL-8, IL-10, and IFN-γ when the patients developed septic shock, indicating that TLR and MDA-5 recognition of HRV might play a crucial role in pathogenesis.

The patient suffered a series of digestive symptoms, such as hepatitis and pancreatitis. However, rhinovirus is rarely associated with these symptoms and we could not get the biopsy of liver and pancreas to find out if virus replicated in these organs. In addition, the patient experienced refractory diarrhoea, and we observed diffuse inflammation in her gastrointestinal tract. Mostly, HRV is liable to acidic environments, and thus, most of the virus is inactivated in the stomach. We only detected 9 reads of rhinovirus RNA in anal swab samples by mNGS, which implied that there was no significant virus replication in the gastrointestinal tract and that diarrhoea was unlikely to be caused by the virus directly. Multiple organ dysfunction syndrome (MODS) is commonly secondary to severe sepsis or septic shock. In shock status, redistribution of blood, a mechanism of our body to regulate the blood of “unnecessary” organs to maintain perfusion of the brain and heart, can lead to gastrointestinal hypoperfusion and dysfunction. Imbalance of intestinal bacterial flora can occur subsequent to MODS and the use of broad-spectrum antibiotics, which results in disorder of gastrointestinal function and severe diarrhoea. The success of faecal microbiota transplantation treatment in patients suffering severe diarrhoea after MODS has proven that gut microbiota imbalance is a possible aetiology of refractory diarrhoea in these patients [24, 25]. Altogether, we presume that the digestive symptoms including hepatitis, pancreatitis and persistent diarrhoea in our patients might be secondary to viral sepsis instead of direct virus insults.

In most scenarios, neurotropic viruses reach the CNS through the bloodstream. Although the blood brain barrier (BBB), which is highly selectively semipermeable, constricts the virus to invade the CNS, in pathological conditions such as virus infection or coinfection of bacteria, meningitis, autoimmune encephalomyelitis, and multiple sclerosis [26], BBB integrity can be compromised, which enables the virus to injure the CNS. However, in our case, the mNGS results of blood samples did not support the diagnosis of viremia. Other underlying mechanisms include the Trojan horse route [27], by which the virus hijacks peripheral circulating immune cells to carry them into the CNS, and the retrograde axonal transport route [28]. HRV was not detectable in the patient’s CSF, but we could not rule out the possibility of an earlier insult by HRV causing the current symptoms. Given the patient’s clinical symptoms, laboratory findings and mNGS reports, we suggested that HRV was the only pathogen accounting for neurological complications and viral sepsis that led to multiorgan dysfunction.

Viral and host factors are associated with disease severity in viral infection. Immunocompromised patients are more susceptible to virus with lower native virulence and prone to severe infection [29, 30]. Early age is also a risk factor for severe viral infection [20]. Neonates and young infants are at high risk of severity. Although the BBB is functionally and morphologically mature in early development, the cellular barrier function and intracellular transfer route of the BBB might be different between early-age children and adults [31]. In reported HRV-related CNS cases, the majority of patients are < 3 years old [9–11]. However, our patient was an immunocompetent 10-year-old girl with no noteworthy medical records or drug use responsible for immunosuppressant status. Whole-exome sequencing did not show any mutations associated with the clinical presentation.

For viral factors, type HRV-45 is a rarely reported virus that was denoted as rhinovirus A clade D. All documented studies about HRV-A45 were associated with acute upper respiratory tract infections [13, 14]. Notably, amino acid mutations in the BC and DE loops of VP1, puff region (EF loop) of VP2, and knob regions of VP3 are known for major neutralization sites in picornavirus [15]. Previous studies have shown that mutations in these sites can change the virulence of viruses [15, 32–34]. We identified 51 missense mutations in our strain. Two unreported amino acid mutations were found in the P2-A and P3-C regions. Additionally, we observed two mutations in the BC loop of VP1 and the EF loop of VP2. Further studies are needed to investigate whether these mutations are related to virulence and facilitate neurotropism.

In summary, we present a case of severe HRV infection that led to CNS involvement and multiorgan dysfunction. Our findings emphasize that HRV can be an underlying aetiology of neurological complications and viral sepsis. In clinical practice, early recognition of the neurological complications of HRV infection and viral sepsis is indispensable for initiating effective treatments to reduce mortality and improve prognosis.

## Data Availability

The datasets supporting the conclusions of this article are included within the article.

## Abbreviations

HRV: Human rhinovirus
CNS: Central nervous system
mNGS: Metagenomics next-generation sequencing
PCT: Procalcitonin
NCPAP: Nasal continuous positive airway pressure
CSF: Cerebrospinal fluid
CRP: C-reaction protein
CRRT: Continuous renal replacement therapy
HEV: Human enterovirus
CPE: Cytopathic effect
BBB: Blood–brain barrier
TLRs: Toll-like receptors
MDA-5: Melanoma differentiation-associated gene 5
MODS: Multiple organ dysfunction syndrome
